# Genome-Wide Association Study and Subsequent Exclusion of *ATCAY* as a Candidate Gene Involved in Equine Neuroaxonal Dystrophy Using Two Animal Models

**DOI:** 10.3390/genes11010082

**Published:** 2020-01-10

**Authors:** Erin N Hales, Christina Esparza, Sichong Peng, Anna R Dahlgren, Janel M Peterson, Andrew D Miller, Carrie J Finno

**Affiliations:** 1 Department of Population, Health and Reproduction, University of California, Davis School of Veterinary Medicine, Davis, CA 95616, USA; enburns@ucdavis.edu (E.N.H.); cnesparza@ucdavis.edu (C.E.); scpeng@ucdavis.edu (S.P.); adahlgren@ucdavis.edu (A.R.D.); janel.peterson@bcm.edu (J.M.P.); 2 Department of Biomedical Sciences, Cornell University College of Veterinary Medicine, Ithaca, NY 14853, USA; andrew.miller@cornell.edu

**Keywords:** alpha-tocopherol, Cayman Ataxia, equine degenerative myeloencephalopathy, genetics, horse, vitamin E

## Abstract

Equine neuroaxonal dystrophy/equine degenerative myeloencephalopathy (eNAD/EDM) is an inherited neurodegenerative disorder of unknown etiology. Clinical signs of neurological deficits develop within the first year of life in vitamin E (vitE) deficient horses. A genome-wide association study (GWAS) was carried out using 670,000 SNP markers in 27 case and 42 control Quarter Horses. Two markers, encompassing a 2.5 Mb region on ECA7, were associated with the phenotype (*p* = 2.05 × 10^-7^ and 4.72 × 10^-6^). Within this region, caytaxin (*ATCAY*) was identified as a candidate gene due to its known role in Cayman Ataxia and ataxic/dystonic phenotypes in mouse models. Whole-genome sequence data in four eNAD/EDM and five unaffected horses identified 199 associated variants within the ECA7 region. MassARRAY^®^ genotyping was performed on these variants within the GWAS population. The three variants within *ATCAY* were not concordant with the disease phenotype. No difference in expression or alternative splicing was identified using qRT-PCR in brainstem across the *ATCAY* transcript. *Atcay^ji^*^-hes^ mice were then used to conduct functional analysis in a second animal model. Histologic lesions were not identified in the central nervous system of *Atcay^ji^*^-hes^ mice. Additionally, supplementation of homozygous *Atcay^ji^*^-hes^ mice with 600 IU/day of dl-α-tocopheryl acetate (vitE) during gestation, lactation, and adulthood did not improve the phenotype. *ATCAY* has therefore been excluded as a candidate gene for eNAD/EDM.

## 1. Introduction

Equine neuroaxonal dystrophy/equine degenerative myeloencephalopathy (eNAD/EDM) is an inherited neurodegenerative disease in horses [1,2,3,4] caused by retrograde degeneration of ascending sensory neurons within the spinocerebellar tracts [5,6]. Current studies support an autosomal dominant with incomplete penetrance, or polygenic mode of inheritance for eNAD/EDM [2]. Clinical signs of eNAD/EDM include an abnormal stance at rest and a symmetric proprioceptive ataxia. The proprioceptive ataxia is worsened when the head is elevated, with hypermetria or pacing often observed [3,5]. Due to the proprioceptive deficits, eNAD/EDM horses are unsafe to ride. Decreased serum vitamin E (vitE) levels have been described in affected horses within the first year of life [3,5]. Supplementation with high doses of vitE, specifically α-tocopherol, decreases disease frequency and severity within affected herds [1]. Currently, diagnosis of eNAD/EDM is difficult as it requires postmortem evaluation of the brainstem and spinal cord. Current unpublished work performed by our group determined the prevalence of eNAD/EDM within horses that underwent a necropsy for spinal ataxia at UC Davis over a 12-year period. Across breeds, equine NAD/EDM was the second most likely diagnosis for spinal ataxia. The most common diagnosis was cervical vertebral compressive myelopathy. Investigation of the genetic etiology of eNAD/EDM could lead to a genetic test whereby susceptible horses could be administered high doses of vitE, in the form of α-tocopherol during gestation and early life [7]. Additionally, genetic tests can be used to make informed breeding decisions, thereby lowering the incidence of disease.

The eNAD/EDM phenotype is similar to ataxia with vitamin E deficiency (AVED) previously described in humans. This disease is caused by a genetic mutation in a vitE transport gene, α-tocopherol transfer protein (*TTPA*) [8]. Previous work in horses has used known human diseases and experimental mouse models of ataxia to exclude the most likely candidate gene, *TTPA* [2]. To identify candidate loci, a previous genome-wide association study (GWAS) was attempted using the Illumina 54 K Equine single nucleotide polymorphism (SNP) array, but relatedness of the affected individuals and low marker density obscured the results [9]. The recent development of the higher density equine 670 K genotyping array [10], in conjunction with development of the Genome-wide Efficient Mixed Model Analysis (GEMMA) [11], has allowed for higher marker density, correction due to relatedness, and successful genome-wide mapping of eNAD/EDM in this study. Within the identified associated region, *ATCAY* was identified as a likely candidate gene and prioritized for further investigation.

ATCAY is a protein which is highly expressed in neurons and has spontaneous mutations known to cause Cayman Ataxia in humans and ataxia with dystonia in three mouse models [12]. Of these, only the hesitant mouse, *Atcay^ji^*^-hes^, can survive postnatal development [13]. Caytaxin, the protein product of *ATCAY*, has three domains: (1) kinesin binding for cellular transportation [14], (2) BNIP-2 and Cdc42GAP homology (BCH), which regulates cell growth, apoptosis, migration, differentiation, and morphogenesis [15], and (3) cellular retinal and the TRIO guanine exchange factor (CRAL-TRIO) known to bind small lipophilic molecules. The CRAL-TRIO domain has been hypothesized to bind a small molecule as polar or more polar than vitE [12].

Despite the hypothesis that caytaxin interacts with vitE, previous research evaluated *Atcay*^hes/hes^ mice fed a vitE supplemented diet, in the form of α-tocopherol, for one month at an unknown life stage [12]. As the ataxic phenotype in the *Atcay*^ji-hes^ mouse develops by postnatal day 14, it was postulated that supplementation would be required during gestation, lactation, and into adulthood in order to prevent clinical signs. Comparison of *Atcay*^hes/hes^ and *Atcay*^hes/+^ individuals has shown differences in protein level, but phenotypic differences have not been explored [16]. Previously, gait analysis of *Atcay*^hes/hes^ and *Atcay*^+/+^ mice was performed through manual measurements [17] and rotarod [12,16]. While considering *ATCAY* as a candidate gene for eNAD/EDM, we additionally sought to refine the phenotyping of the three *Atcay^ji^*^-hes^ murine genotypes with Treadscan [18], horizontal ladder beam [19], and balance beam testing.

In this study, we conducted a GWAS using the new 670 K SNP array with the hypothesis that a gene or genes involved in vitE pathways would be identified in genome-wide associated regions. Additionally, we hypothesized that the *Atcay*^ji-hes^ hesitant mouse phenotype would be modulated with supplementation with high doses of vitE during gestation, lactation, and postnatal development.

## 2. Materials and Methods

### 2.1. Genome Wide Association Study

DNA previously collected was used to genotype 69 Quarter Horses (27 cases, and 42 controls) using the Axiom Equine Genotyping Array (Axiom MNEC670, Affymetrix, Santa Clara, CA, USA). All case and control horses were naturally raised and selected based on owner reports that the animals were without pasture access, and therefore likely vitE deficient, throughout the first two years of life. Affymetrix Axiom Analysis Suite (version 2.0 Santa Clara, CA, USA) was used to generate plink [20] compatible tfiles. All horses had a genotyping rate above 90%. SNPs that had a genotyping rate below 95% or a minor allele frequency below 5% were filtered out, leaving 504,035 markers. Additionally, all SNP locations were converted to EquCab3.0 using the publicly available conversion comma separated value file [21]. Any SNP on an unplaced contig, or without a unique location in EquCab3.0 was removed from analysis. After filtering and conversion, 488,210 SNPs remained in all 69 horses for analysis. A preliminary chi-square allelic association test was run using plink [20] to assess population stratification. Following this, a genome-wide efficient mixed-model analysis (GEMMA) [11] was performed using option 1 with a Wald test. Graphs were generated using the qqman [22] and MultiMeta [23] packages in Rstudio (Boston, MA, USA). All GWAS test were run using EquCab3.0 remapped locations from the publicly available file [21].

### 2.2. Candidate Gene Identification

After the 2.5 megabase (Mb) associated region (≈2.0–4.5 Mb) on equine chromosome (ECA) 7 was identified in EquCab3.0, 147 candidate genes were evaluated within the region (Table S1). Using PANTHER gene ontologies [24], priority was assigned to genes involved in vitE metabolism or nervous system processes. *ATCAY* was identified as a highly plausible candidate gene due to its causative role in cerebellar ataxia of people in the Cayman Islands [12]. Additionally, there are three mouse models and a rat model with spontaneous mutations in the *Atcay* gene that have ataxic phenotypes [12,25].

### 2.3. Putative Causal Variant Genotyping

Whole-genome sequence (WGS) data was generated from nine Quarter Horses (*n* = 4 postmortem confirmed cases and *n* = 5 postmortem confirmed controls). Paired-end reads (150 bp paired end) were generated on an Illumina HiSeq 2000 and are available under accession PRJNA526073. Reads were trimmed using fastqc (Babraham Institute, Cambridge, UK) and mapped to the EquCab 3.0 NCBI reference using bwa-mem [26]. An average of 12× coverage was achieved. Variants from this WGS data were identified using FreeBayes [27]. Within the 2.5 Mb associated region, 874 variants were identified across all individuals. Variants were filtered to a *p* < 0.05 based on an allelic Fisher’s Exact test using SnpSift [28], and predicted effects of variants were called using SNPeff [29]. From this set of associated variants, variants with the smallest *p* value (i.e., *p* = 0.07) were further evaluated for prioritization and genotyping in the entire GWAS population (*n* = 69 horses). No correction for multiple testing was performed due to the small samples size and to ensure putative variants were not excluded. Significant variants were prioritized based on annotations and putative effects using the NCBI annotation of EquCab3.0, as well as putative haplotype structure. Additionally, any variants identified within *ATCAY* were included. In total, 200 variants were identified as unique to a haplotype block, or as having a high or moderate predicted effect on protein function. MassARRAY assay probes were able to be designed for 199 of these variants.

The additional variants were genotyped using the MassARRAY^®^ platform (Agena Bioscience, San Diego, CA, USA) through Neogen Corporation (Lincoln, NE, USA). The 69 Quarter Horses used in the GWAS were genotyped on this platform. Twenty-three variants were filtered out via QC analysis in plink [20]; 10 failed genotyping and the other 13 had minor allele frequencies below 5%. The GWAS SNPs were then combined with the MassARRAY variants for analysis in GEMMA [11]. The correlation filter in GEMMA resulted in the loss of another 10 SNPs due to a correlation with the relationship matrix (*r*^2^ > 0.9999). In total, 166 SNPs on ECA7 were tested alongside the original GWAS SNPs using the Wald test in GEMMA [11] with a centered relatedness matrix. It was hypothesized that causal variants would be more perfectly concordant with phenotype than those associated variants from the GWAS.

### 2.4. ATCAY Expression Quantification

Quantitative Reverse Transcription Polymerase Chain Reaction (qRT-PCR) was performed across the equine *ATCAY* transcript in brainstem tissue from 15 additional horses (eight cases and seven controls) (Table S2). Disease status was postmortem confirmed in all these horses, as previously described [3]. Primers were designed, using Primer3Plus [30], to the EquCab3.0 sequence of *ATCAY* spanning exons 1–2, 2–3, 5–6, 8–9, and 10–12. *HPRT1* was used as the housekeeping gene as previously described [31]. RNA was extracted from equine brainstem using a Trizol^®^ chloroform separation and column clean up using Direct-zol™ RNA miniprep Plus kit from Zymo Research (Irvine, CA, USA). DNA digestion was performed on column with the provided DNase I. Next, cDNA was synthesized using Superscript III^®^ from Thermofisher (Waltham, MA, USA) according to the manufacturer’s instructions. Transcript quantification was carried out using Brilliant III Ultra-Fast SYBR qPCR Master Mix from Agilent (Santa Clara, CA, USA) on an AriaMX machine. All samples were run in triplicate and the ΔCq averaged for analysis. Thresholds were standardized for each primer set.

### 2.5. Rederived *Atcay^ji-hes^* Mice

The *Atcay^ji-hes^* mouse was embryonically rederived from Jackson Laboratories. One generation of backcrosses was generated using C3He/J mice (Catalog #000659, Jackson Laboratories, Bar Harbor, ME, USA). The offspring of the F1 generation were maintained on a basal diet with sufficient vitE (35 mg of dl-α-tocopheryl acetate/kg feed) to recapitulate the phenotype. Once recapitulated, a subset of breeding pairs was placed on a vitE supplemented diet (vitE+++ = 600 mg of dl-α-tocopheryl acetate/kg feed) at introduction of the male and maintained on this diet throughout gestation and lactation. This diet has been previously demonstrated to prevent the ataxic phenotype in tocopherol-transfer protein-null mice [32]. Once weaned, these mice were maintained on the vitE+++ diet until terminal use at one or two months of age. All murine protocols were approved by the Institutional Animal Care and Use Committee (IACUC) protocol #19920.

### 2.6. *Atcay^ji-hes^* Genotyping

Using the previously identified IAP element insertion [12], Primer3Plus [30] was used to design primers whose products span the insertion site, with the mutant primer covering the insertion site to allow for differentiation. The primer design was modified from previously published protocols [12,33]. The previously published common forward primer was used and two reverse primers were designed: F) CCTCCCTGCACAGACACAATAG, R_WT_) GGGATGTTAGGGTTTACCACCA, and R_hes_) GCTTCCCACAGATGACAAGG. Genotyping PCR was performed with standard AmpliTaq Gold (Cat. No. N8080244) and the accompanying PCR buffer I from Applied Biosystems (Foster City, CA). The temperature program used was five-minute denaturation, followed by 30 amplification cycles with 61 °C annealing temperature and a five-minute clean-up phase at 72 °C. Products were visualized either on a 1.5% agarose gel with SYBR^®^ Safe (Cat. No. S33a02 from Invitrogen, Carlsbad, CA, USA), or a QIAxcel advanced using the 15 bp-1Kb alignment marker and 50–800 bp size marker (Qiagen Inc. Hilden, Germany). The wild type allele yields a 332 bp product while the mutant allele yields a 293 bp product.

### 2.7. *Atcay^ji-hes^* Phenotyping

As the *Atcay^ji-hes^* phenotype can be visually determined [17], we evaluated methods to quantify the obvious gait abnormalities. Balance beam, TreadScan [18,34], and horizontal ladder beam [19,32] tests were used to measure balance and coordination of movement. The *Atcay*^hes/hes^ mice were unable to complete any portion of the balance beam test due to their severe ataxic and dystonic phenotype. For this reason, only the *Atcay*^hes/+^ and *Atcay*^+/+^ individuals were scored on the balance beam. The balance beam test was run on the two genotypes at one and two months of age, with one-month individuals being repeatedly tested at two months of age. The Treadscan [18,34] and horizontal ladder test were only evaluated at the endpoint for each mouse regardless of genotype. Treadscan was performed at 6 m/s due to the inability of the *Atcay*^hes/hes^ one-month mice to maintain any higher treadmill speeds.

### 2.8. Statistical Analysis

All phenotyping test results were analyzed using Prism 8 (GraphPad, San Diego, CA, USA). Normality was assessed using a Q-Q plot and Shapiro–Wilk test. Non-normally distributed data was log_10_-transformed when possible. Any data that could not be normalized was analyzed using a Kruskal–Wallis test (balance beam slips and horizontal ladder beam rear foot faults). All data that could be normalized was analyzed using a one-way ANOVA (TreadScan and horizonal ladder beam) or mixed model analysis (balance beam). Multiple comparisons were carried out using Tukey’s post-hoc comparisons. Significance was set at *p* < 0.05.

### 2.9. Central Nervous System Histopathology

Thirty-one mice were histologically evaluated for abnormalities of the central nervous system (CNS). Two mice of each sex (male or female) on each diet (basal or vitE+++) of each genotype (*Atcay*^hes/hes^ or *Atcay*^+/+^) at each time point (one-month or two-month) were evaluated (Table S3). One individual was not able to be collected, a female one-month *Atcay*^hes/hes^ on the vitE+++ diet. At one or two months of age, mice were euthanized using 60 mg/kg of Fatal-Plus^®^ C IIN (Vortech Pharmaceuticals, Dearborn, MI, USA) via intraperitoneal injection. Lack of sensation was verified by checking for corneal and withdrawal reflexes. Mice were then perfused with 10 mL of phosphate buffered saline (PBS) followed by 10–15 mL of 4% paraformaldehyde (PFA). After fixation, the spinal column and brain case were excised, cleaned of muscle and connective tissue, placed in a 15 mL conical tube, and covered with 4% PFA. The PFA was exchanged for 70% ethanol between one and three days after perfusion. Once all genotypes and diets were collected, the central nervous tissues were sectioned and evaluated by a veterinary pathologist (ADM) following an additional two days of fixation in formical for decalcification purposes (StatLab, McKinney, TX, USA). Additionally, ionized calcium binding adaptor molecule 1 (Iba1) (catalog #019–19741 Wako Chemicals, Richmond, VA, USA) immunohistochemical staining was performed on four *Atcay*^hes/hes^ and four *Atcay*^+/+^ mice as previously described [34]. Serial sections were also assessed for myelin pathology via Luxol fast blue histochemical staining and axonal pathology via Bielschowsky histochemical staining.

## 3. Results

### 3.1. Genome-Wide Association Study

**Figure 1:**
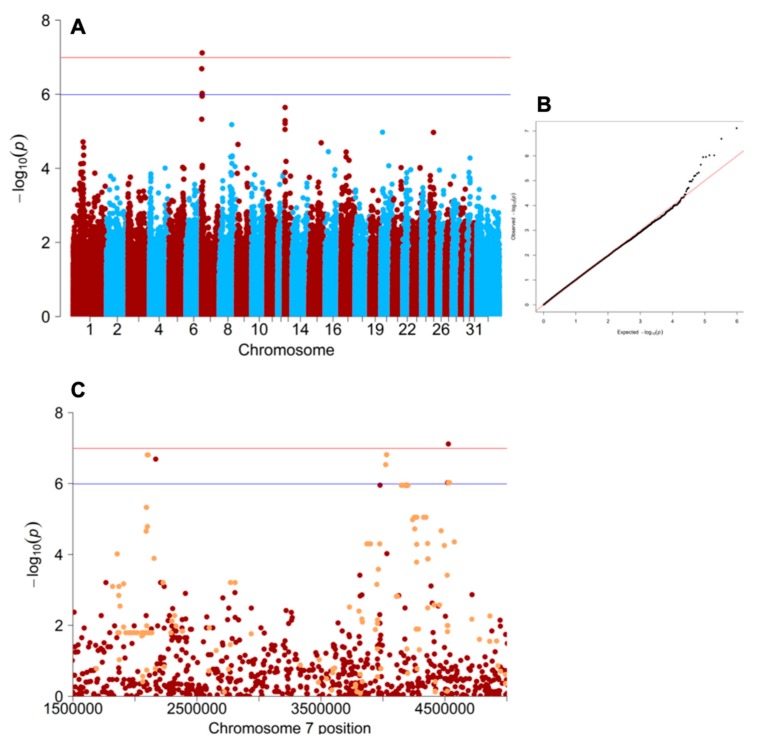
A 2.5 Mb region on ECA7 is associated with the equine neuroaxonal dystrophy/equine degenerative myeloencephalopathy (eNAD/EDM) phenotype. (**A**) Manhattan plot of the original genome-wide association study (GWAS) results showing the associated region on ECA7. The red line indicates Bonferroni corrected significance (*p* = 1.024 × 10^-7^ and -log(*p*) = 6.99), and the blue line represents and 10% False Discovery Rate (FDR) correction (*p* = 1.024 × 10^-6^ and -log(*p*) = 5.99). (**B**) Q-Q plot showing a low likelihood of false positives or negatives. (**C**) Manhattan plot of ECA7 with GWAS data in red and variants from the MassARRAY^®^ data highlighted orange. Red and blue lines have same values as in (A). All data are listed by their EquCab3.0 positions.

Evaluation of population stratification in plink [20] using the genomic inflation value (lambda) from a chi-square basic allele test was 2.1. When GEMMA [11] was used to create a centered relatedness matrix and a Wald association test was performed, the genomic inflation was reduced to 1.08. Visualizing the result of the Wald test from the mixed linear model analysis revealed only a single SNP on ECA7 (AX-104596120, EquCab3.0 location ECA7: 4,528,348) that surpassed Bonferroni genome-wide significance (*p* < 1.024 × 10^−7^ Figure 1A,B). Further exploration of this region identified an additional three SNPs that achieved suggestive genome wide significance, with a false discovery rate correction of 10% (Figure 1A). These four SNPs encompassed a 2.5 Mb (≈2.0–4.5 Mb) region on ECA7 in EquCab3.0. The equine *ATCAY* homolog is centered between the associated SNP markers (EquCab3.0; ECA7: 2,752,177–2,779,255) using the NCBI reference annotation for EquCab3.0. The EquCab2.0 and EquCab3.0 SNP and top candidate gene positions are listed in Table S4.

### 3.2. Equine *ATCAY* Genotyping

WGS data mapped to EquCab3.0 from horses with known eNAD/EDM neurological status were used to identify associated variants within the 2.4 Mb associated region on ECA7. Three of the variants identified were within *ATCAY*. The first (EquCab3.0; ECA7: 2,755,141 g. C > T) was annotated as a synonymous change by SNPeff based on the NCBI annotation (www.ncbi.nlm.nih.gov/genome/annotation_euk/Equus_caballus), and verified in the ENSEMBL annotation (https://uswest.ensembl.org/Equus_caballus/Info/Annotation). The second variant (EquCab3.0; ECA7: 2,769,147 g. C > T), and third variant (EquCab3.0; ECA7: 2,770,991 g. A > T) were annotated to be within introns 7 and 10, respectively. The second variant was within 120 bp of exon 8. Genotyping results from the additional 166 variants (Table S5) that passed QC, including these three *ATCAY* variants, were added to the 488,210 GWAS SNPs and evaluated using GEMMA (Figure 1C). None of the variants in the *ATCAY* region were more perfectly concordant with the phenotype than the most concordant GWAS SNPs (Figure 1C). There was a set of eight SNPs that passed 10% FDR correction identified at 2.1 Mb within an ambiguously annotated *ZNF77* transcript (*LOC100059925* Table S5).

### 3.3. Equine *ATCAY* Expression Quantification

**Figure 2:**
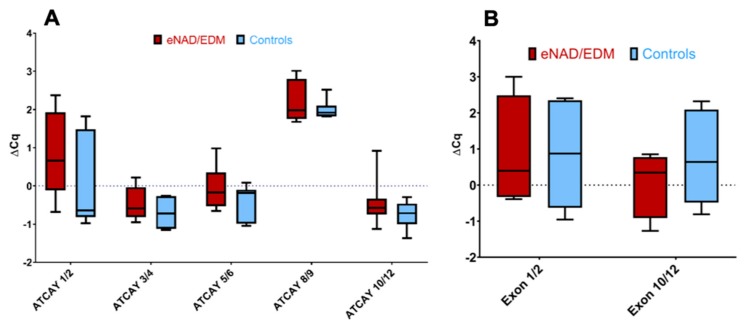
*ATCAY* expression does not differ between confirmed eNAD/EDM horses and controls. (**A**) Each of the five exon pairs tested showed no significant differences between the eight postmortem confirmed cases and seven postmortem confirmed controls. (**B**) Standardization of the cycle threshold (*n* = 5 cases and *n* = 4 controls) between exons 1 and 12 showed no evidence for alternative splicing across the gene.

The *ATCAY* mRNA annotation contains 13 exons. Expression of the transcript was not different between eNAD/EDM horses (*n* = 8) and control horses (*n* = 7) across the 10 exons tested (Figure 2A). To confirm that there was no evidence of alternative splicing between exons 1 and 10, nine horses (*n* = 4 eNAD/EDM cases and *n* = 5 controls) were assayed again with a standardized cycle threshold. There was no evidence of differential expression between exon 1 and exon 10 (Figure 2B). Therefore, alternative splicing at *ATCAY* was deemed unlikely.

### 3.4. Phenotype of *Atcay^ji-hes^* Mice

On the Treadscan, *Atcay*^hes/hes^ mice demonstrated difficulty in maintaining their pace, even at the slow 6 m/s setting. This led to inconsistencies in the Treadscan measurements and exclusion of large numbers of individuals. Although *Atcay*^hes/hes^ mice have a rear limb ataxia and dystonia, the analysis software could not quantify this abnormal gait in the few animals that were able to be measured (data not shown). Due to all these factors, the Treadscan data was deemed uninformative in the evaluation of this ataxic and dystonic phenotype of *Atcay*^ji-hes^ mice.

The balance beam and horizontal ladder beam tests were more reliable, with consistent measurements upon repeated evaluations. Balance beam measurements were not collected on the *Atcay*^hes/hes^ mice due to their severe phenotype. *Atcay*^he*s*/+^ mice were found to have no significant differences from *Atcay*^+/+^ in crossing time (Figure S1) or slips (data not shown) at any difficulty on the balance beam. Additionally, comparison of the *Atcay*^hes/+^ and *Atcay*^+/+^ mice revealed no differences in horizontal ladder beam crossing time or rear foot faults (data not shown). Therefore, *Atcay*^hes/+^ mice were excluded from the remaining analysis of horizontal ladder beam tests.

**Figure 3:**
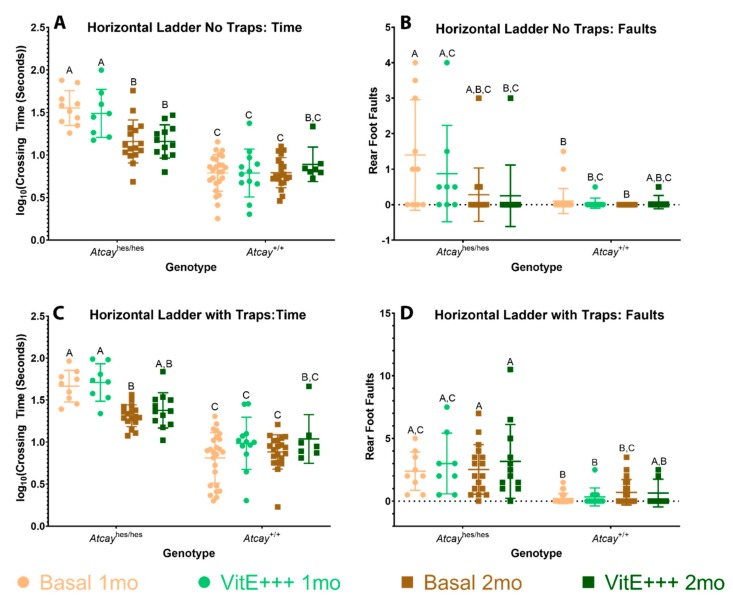
*Atcay*^hes/he*s*^ mice have difficulty crossing the horizontal ladder. Average crossing time (**A**) and rear foot faults (**B**) for the test without traps. Average crossing time (**C**) and rear foot faults (**D**) for the test with four evenly spaced traps. Crossing time typically segregates the genotypes. All groups were compared using Tukey’s post-hoc comparisons. Letters signify significance (*p* < 0.05) between groups.

All homozygous *Atcay*^hes/hes^ mice, regardless of diet, crossed the horizontal ladder significantly slower than *Atcay*^+/+^ mice, with the exception of the two-month *Atcay*^+/+^ vitE+++ mice (Figure 3A, C). *Atcay*^hes/hes^ mice were found to have significantly more foot faults when traps were present as compared to *Atcay*^+/+^ mice at one month of age (Figure 3D). However, without traps, overlap between genotypes and diets was more common. The only major difference was between the one-month *Atcay*^hes/hes^ mice on a basal diet as compared to most of the *Atcay*^+/+^ groups (Figure 3B), with the exception of the *Atcay*^+/+^ two-month supplemented mice. Additionally, the two-month *Atcay*^hes/hes^ mice were significantly faster than their younger counterparts without traps (Figure 3A). This trend holds true with the two-month basal *Atcay*^hes/hes^ mice navigating the obstacle with traps, but not for the supplemented *Atcay*^hes/hes^ mice (Figure 3C). No differences were observed in mice on the vitE+++ diet when compared to their basal counterparts. These findings suggest that the *Atcay*^hes/hes^ phenotype improves with age.

### 3.5. *Atcay^ji-hes^* Histology

A total of 31 mice were fixed in 4% paraformaldehyde (PFA) and examined histologically using hematoxylin and eosin staining with light microscopy. No evidence of histologic lesions within the cerebrum, brainstem, cerebellum, or spinal cord were noted. Additionally, slides from one-month *Atcay*^hes/hes^ (*n* = 4) mice on a basal diet compared to age, diet, and sex-matched *Atcay*^+/+^ (*n* = 4) mice demonstrated no increase in immunoreactivity to Iba1, a marker of microglial activity. Myelin and axonal abnormalities were not noted with Luxol fast blue or Bielschowsky histochemical stains, respectively.

## 4. Discussion

A 2.5 Mb region on ECA7 was identified as associated with eNAD/EDM in a strictly phenotyped population of Quarter Horses. *ATCAY* was identified within this region as a plausible candidate gene. However, examining whole-genome sequencing data from four affected horses did not identify an obvious causal variant. Further, expression of *ATCAY* within the brainstem of affected horses was not altered and evaluation of *Atcay*^ji-hes^ mice demonstrated that the hesitant phenotype improves over time, but vitE supplementation has no effect. This is a stark contrast to the ability of vitE to prevent the phenotype when sufficiently high doses are administered in *Ttpa*^−/−^ mice and eNAD/EDM horses [32]. The inability to prevent the phenotype with vitE provides indirect support that caytaxin does not bind vitE. Therefore, the hypothesis that genetic variation at the *ATCAY* locus contributes to the eNAD/EDM phenotype is not supported based on evidence from the equine and murine model experiments performed.

GWAS techniques have previously been used in attempts to map eNAD/EDM in equine genome. These efforts encountered issues with population stratification due to relatedness and the multiple breed study design [9]. Small sample size, insufficient marker density, and unknown environmental factors were also likely contributors to these studies being underpowered. Since then, additional research has demonstrated that a vitE deficiency during the first year of life is necessary to develop the eNAD/EDM phenotype [35]. To control for this now established environmental variable, the current study only included horses raised without access to fresh green pasture and without administration of supplemental vitE during the first two years of life. Additionally, the population was limited to Quarter Horses to control for any possible genetic heterogeneity across breeds. These breed and environmental restrictions allowed for the successful mapping of the eNAD/EDM phenotype to a 2.5 Mb region on ECA7. Previous pedigree-based work has suggested eNAD/EDM is inherited in an autosomal dominant or incompletely dominant fashion [1,2]. However, MassARRAY^®^ genotyping within the associated region showed strong association at both 2.0 and 4.2 Mb, which may support an additive inheritance pattern with multiple loci contributing to disease. Additionally, as linkage disequilibrium can extend over large regions within breeds of horses, these two markers could be representing one functional variant within the region. Future research should use quantitative trait loci mapping to explore the ECA7 region along with the region on ECA13 that only approaches an FDR correction for multiple testing.

*ATCAY*, which encodes caytaxin, was prioritized as a candidate gene due to its predicted protein structure [12], and known association with many ataxic phenotypes [12,13,16]. The protein structure of caytaxin has sequence similarity with tocopherol transfer protein-α, the enzyme known to selectively bind a vitamer of vitE in the liver and package it for transport around the body. This enzyme is encoded by *TTPA,* which has been evaluated and excluded as a candidate gene for eNAD/EDM [2]. However, both humans and mice have similar phenotypes due to a *TTPA/Ttpa* deficiency, including an early age of onset of generalized symmetric ataxia, decreased circulating and tissue vitE levels, and stabilization of clinical signs after a certain age [2]. Two mutations have been found in *ATCAY* that completely segregate with Cayman Ataxia in humans [12]. In animal models, the jittery, sidewinder, and jittery-hesitant mouse as well as the dystonic rat all have spontaneous mutations in *Atcay* leading to reduced or aberrant transcripts and a range of ataxic and dystonic phenotypes [12,25]. Interestingly, all the rodent models are unable to survive past five weeks of age with the exception of the *Atcay*^ji-hes^ mouse [25]. The hesitant gait characteristic of the *Atcay*^ji-hes^ mouse also resembles the hypermetric and spastic gait of ataxic horses with eNAD/EDM [3]. It was postulated that, due to the protein sequence homology, *ATCAY* could be responsible for binding vitE within the central nervous system [12]. This possibility, combined with the phenotypic characteristics of the hesitant mouse, made *ATCAY* a plausible candidate gene.

In the current study, *ATCAY* was excluded as a likely candidate for eNAD/EDM based on genetic and functional assays. Three variants were identified within *ATCAY*, but they did not associate with the disease phenotype, and none were predicted to have a deleterious effect on protein function. Analysis of *ATCAY* expression within the brainstem of eNAD/EDM-affected horses also showed no difference in expression; however, protein levels were not evaluated. There was no support for the presence of alternative transcripts using qRT-PCR. According to the ENSEMBL annotation of EquCab3.0, there are two predicted transcripts of *ATCAY* with alternative 3’ UTRs; however, the NCBI annotation contains only one transcript. Alternatively spliced transcripts have been identified in both the human and mouse homologues [12,16]. Therefore, further work is needed to clarify the presence or absence of alternative splicing in the horse. Protein expression of *ATCAY* in eNAD/EDM affected horses should also be considered as previous work has shown that caytaxin protein expression changes significantly throughout the first six months of a mouse’s life [16], and changes in relative transcript abundance do not always mimic protein levels [33].

In addition to the lack of associated genetic variants or transcript expression changes in *ATCAY*, no histologic lesions supportive of neurodegeneration were identified within the central nervous system of *Atcay*^ji-hes^ mice. It is likely that the phenotypic changes in the hesitant mouse are due to subcellular changes of neurite growth within the cerebellum [17,36,37]. Prevention of the hesitant phenotype was attempted through vitE supplementation but was unsuccessful. Regardless of diet, the neurobehavioral phenotype of hesitant mice was observed to improve over time. Additionally, vitE supplementation of *Atcay*^+/+^ mice at two months of age caused the average crossing times to be similar to mutant, which was not true of the other *Atcay*^+/+^ groups. It is postulated that this is due to the small group size (*n* = 7) and presence of an outlier within the vitE+++ two-month *Atcay*^+/+^ group. The inability of vitE supplementation to improve the phenotype of *Atcay*^hes/hes^ mice provides indirect support that caytaxin does not bind vitE [12] and that *ATCAY* is not related to the eNAD/EDM phenotype. It has been proposed that neuronal changes within the *Atcay*^ji-hes^ mouse are subcellular, altering cholinergic machinery axonal transport without causing neuronal degradation [36]. Additional work has also shown that caytaxin binds to kinesin lite chain for transport of ATP citrate lyase towards neurite terminals, leaving no room for binding of vitE [36].

Two additional genes, Zinc Finger Protein 77 (*ZNF77*) and Acyl-CoA Synthetase Bubblegum Family Member 2 (*ACSBG2*), are located within the eNAD/EDM associated region on ECA7 and have been prioritized for further analysis based on genomic location a functional plausibility (Table S1). *ZNF77* encodes a ubiquitously expressed transcription factor of yet unclarified importance. However, it has been suggested to have a role in vesicle trafficking and extracellular matrix regulation [38]. *ACSBG2* is involved in fatty acid synthesis and is expressed solely in testicular and brainstem tissues [39]. The variants in these genes as well as their expression are currently being evaluated.

Limitations of the current study include restricting the equine genomic association to Quarter Horses, as eNAD/EDM occurs across breeds [4,40,41]. However, it is possible that the genetic cause of eNAD/EDM is heterogenous and varies by breed. Further work would be needed to elucidate the plausible causes of eNAD/EDM in other breeds as well as to find the mutation underlying this genomic association. This could be done through additional GWA studies, or by genotyping additional horses for the ECA7 haplotype. Second, although mice in this study were supplemented with 600 mg of vitE, an increase in circulating or tissue levels was not confirmed. However, previous work in the lab has successfully used the same diet to increase brain tissue levels of vitE in *Ttpa*^−/−^ mice [32].

In conclusion, *ATCAY* was excluded as a candidate gene for eNAD/EDM based on genetic polymorphisms, gene expression, and functional assays using a mouse model. The ataxic and dystonic gait of *Atcay*^ji-hes^ mice was best measured using a horizontal ladder beam apparatus and does not improve with vitE supplementation. Additionally, despite a known difference in protein level [16], *Atcay*^hes/+^ mice have no observable phenotypic abnormalities. Additional work is required to investigate other genes in the region of association on ECA7 as candidate genes for eNAD/EDM in Quarter Horses.

## Supplementary Materials

The following are available online at https://www.mdpi.com/2073-4425/11/1/82/s1, Table S1: Positional candidate genes in GWAS region and their predicted function, Table S2: Equine qRT-PCR cohort, Table S3: Mouse histology cohort, Table S4: Conversion of equine genome coordinates between genome builds, Table S5: Additional variants genotyped within the GWAS associated region, Figure S1: Balance beam data showing no changes for the *Atcay*^ji-hes^ mouse. WGS raw sequenced data available on the SRA database under Accession number PRJNA526073.
