# Supplementary material for: Genome-Wide Association Study and Subsequent Exclusion of ATCAY as a Candidate Gene Involved in Equine Neuroaxonal Dystrophy Using Two Animal Models
Source: Genes (Basel). 2020 Jan 10;11(1):82. doi: 10.3390/genes11010082 (PMC7016928; doi:10.3390/genes11010082)
Supplement: Supplementary file 1 [file genes-11-00082-s001.pdf]

Table S1: All positional candidate genes within the GWAS identified region (2.1-4.5 Mb). An additional 500 Kb were added to each end of the window to ensure no plausible candidate genes were missed. Genes were identified based on the NCBI annotation of EquCab3.0 and ontologies were identified using PANTHER.24 A "-" symbolizes that no information of that type exists for the transcript. Ontology information is based off of the homo sapiens reference as equine specific data was unavailable for the majority of the genes identified.

GENE ID	ECA7 Position	Molecular Function	Biological Process	Protein Class
LMNB2	1,588,826-1,606,484	-	cellular component morphogenesis(GO:0032989)	-
GADD45B	1,620,330-1,622,432	MAP kinase activity(GO:0004707);MAP kinase kinase activity(GO:0004708)	activation of MAPKK activity(GO:0000186);cell cycle(GO:0007049);regulation of cell cycle(GO:0051726)	-
GNG7	1,643,237-1,769,251	G-protein coupled receptor activity(GO:0004930);protein binding(GO:0005515)	-	heterotrimeric G-protein(PC00117)
DIRAS1	1,771,381-1,777,763	GTPase activity(GO:0003924);protein binding(GO:0005515)	G-protein coupled receptor signaling pathway(GO:0007186);I-kappaB kinase/NF-kappaB signaling(GO:0007249);MAPK cascade(GO:0000165);cell adhesion(GO:0007155);cell surface receptor signaling pathway(GO:0007166);chemical synaptic transmission(GO:0007268);intracellular protein transport(GO:0006886);nervous system	small GTPase(PC00208)

			process(GO:0050877);receptor-mediated endocytosis(GO:0006898)	
SLC39A3	1,785,105-1,790,626	metal ion transmembrane transporter activity(GO:0046873)	divalent metal ion transport(GO:0070838);inorganic cation transmembrane transport(GO:0098662);transition metal ion transport(GO:0000041)	transmembrane receptor regulatory/adaptor protein(PC00226)
SGTA	1,802,072-1,819,610	protein-containing complex scaffold activity(GO:0032947)	chaperone-mediated protein folding(GO:0061077);positive regulation of cellular process(GO:0048522);posttranslational protein targeting to endoplasmic reticulum membrane(GO:0006620);protein ubiquitination(GO:0016567);ubiquitin-dependent ERAD pathway(GO:0030433)	-
THOP1	1,819,706-1,841,367	metalloendopeptidase activity(GO:0004222)	peptide metabolic process(GO:0006518)	metalloprotease(PC00153)
ZNF554	1,841,508-1,862,674	-	-	KRAB box transcription factor(PC00029)

LOC111774170	1,846,368-1,850,410	-	-	-
ZNF555	1,866,666-1,879,729	-	-	KRAB box transcription factor(PC00029)
LOC106783345	1,880,203-1,881,424	-	-	-
ZNF556	1,895,154-1,910,671	-	-	KRAB box transcription factor(PC00029)
ZNF14	1,924,597-1,932,741	-	-	-
ZNF77	1,942,392-1,959,392	-	-	KRAB box transcription factor(PC00029)
LOC111774440	1,958,983-1,964,210	-	-	-
ZNF77	1,975,582-1,999,147	-	-	KRAB box transcription factor(PC00029)
ZNF791-like	2,003,236 - 2,016,915	-	-	-
ZNF77	2,028,633 - 2,041,240	-	-	KRAB box transcription factor(PC00029)
LOC111774438	2,043,395 - 2,052,928	-	-	-
ZNF77-like	2,056,618 - 2,069,004	-	-	-
ERVPABLB-1-like	2,070,481 - 2,072,520	-	-	-
ZNF77	2,082,408 - 2,093,768	-	-	KRAB box transcription factor(PC00029)

LOC106783362	2,093,902 - 2,103,628	-	-	-
ZNF77	2,099,335 - 2,151,434	-	-	KRAB box transcription factor(PC00029)
LOC111774441	2,119,303 - 2,139,763	-	-	-
LOC111774442	2,121,577 - 2,122,928	-	-	-
LOC111774439	2,169,699 - 2,171,037	-	-	-
TLE6	2,181,784 - 2,192,256	repressing transcription factor binding(GO:0070491);transcription corepressor activity(GO:0003714)	canonical Wnt signaling pathway(GO:0060070);negative regulation of canonical Wnt signaling pathway(GO:0090090);nucleic acid-templated transcription(GO:0097659)	transcription cofactor(PC00217)
TLE2	2,193,985 - 2,216,212	repressing transcription factor binding(GO:0070491);transcription corepressor activity(GO:0003714)	canonical Wnt signaling pathway(GO:0060070);negative regulation of canonical Wnt signaling pathway(GO:0090090);nucleic acid-templated transcription(GO:0097659)	transcription cofactor(PC00217)
TLE5/AES	2,225,713 - 2,234,007	repressing transcription factor binding(GO:0070491);transcription corepressor activity(GO:0003714)	canonical Wnt signaling pathway(GO:0060070);negative regulation of canonical Wnt signaling pathway(GO:0090090);nucleic acid-templated transcription(GO:0097659)	transcription cofactor(PC00217)
U6 spliceosomal RNA	2,241,121 - 2,241,227	-	-	-

GNA11	2,256,345 - 2,275,859	G-protein coupled receptor activity(GO:0004930);G-protein coupled receptor binding(GO:0001664);GTPase activity(GO:0003924);adenylate cyclase activity(GO:0004016);phospholipase C activity(GO:0004629);protein-containing complex binding(GO:0044877)	action potential(GO:0001508);activation of phospholipase C activity(GO:0007202);adenylate cyclase-modulating G-protein coupled receptor signaling pathway(GO:0007188);cAMP-mediated signaling(GO:0019933);dopamine receptor signaling pathway(GO:0007212);inositol phosphate-mediated signaling(GO:0048016);phospholipase C-activating G-protein coupled receptor signaling pathway(GO:0007200);regulation of adenylate cyclase activity(GO:0045761);regulation of cAMP-mediated signaling(GO:0043949)	heterotrimeric G-protein(PC00117)

GNA15	2,281,724 - 2,300,313	G-protein coupled receptor activity(GO:0004930);G-protein coupled receptor binding(GO:0001664);GTPase activity(GO:0003924);adenylate cyclase activity(GO:0004016);phospholipase C activity(GO:0004629);protein-containing complex binding(GO:0044877)	activation of phospholipase C activity(GO:0007202);adenylate cyclase-modulating G-protein coupled receptor signaling pathway(GO:0007188);cAMP-mediated signaling(GO:0019933);dopamine receptor signaling pathway(GO:0007212);inositol phosphate-mediated signaling(GO:0048016);phospholipase C-activating G-protein coupled receptor signaling pathway(GO:0007200);regulation of adenylate cyclase activity(GO:0045761);regulation of cAMP-mediated signaling(GO:0043949)	heterotrimeric G-protein(PC00117)
S1PR4	2,306,970 - 2,311,110	G-protein coupled receptor activity(GO:0004930)	cell surface receptor signaling pathway(GO:0007166);cell-cell signaling(GO:0007267);intracellular signal transduction(GO:0035556)	G-protein coupled receptor(PC00021)
NCLN	2,313,437 - 2,330,085	-	regulation of signal transduction(GO:0009966);signal transduction(GO:0007165)	-
CELF5	2,337,756 - 2,383,973	mRNA binding(GO:0003729)	alternative mRNA splicing, via spliceosome(GO:0000380);mRNA splice site selection(GO:0006376);regulation of alternative mRNA splicing, via spliceosome(GO:0000381)	-

SMIM24	2,392,488 - 2,395,516	-	-	-
SMIM24-like	2,396,354 - 2,398,898	-	-	-
NFIC	2,404,452 - 2,458,741	RNA polymerase II transcription factor activity, sequence-specific DNA binding(GO:0000981)	negative regulation of transcription by RNA polymerase II(GO:0000122);positive regulation of transcription by RNA polymerase II(GO:0045944);transcription by RNA polymerase II(GO:0006366)	nucleic acid binding(PC00171);transcription factor(PC00218)
DOHH	2,494,342 - 2,508,424	-	-	-
FZR1	2,507,133 - 2,529,417	protein binding(GO:0005515)	cell cycle(GO:0007049);proteolysis(GO:0006508)	enzyme modulator(PC00095)
C7H19orf71	2,531,010 - 2,540,978	-	-	-
MFSD12	2,541,111-2,549,382	-	organic substance transport(GO:0071702)	-
HMG20B	2,556,256-2,561,752	-	-	-
GIPC3	2,563,658-2,570,396	acetyltransferase activity(GO:0016407)	cellular process(GO:0009987)	acetyltransferase(PC00038)

TBXA2R	2,570,729-2,582,573	G-protein coupled receptor activity(GO:0004930);adenylate cyclase activity(GO:0004016)	activation of adenylate cyclase activity(GO:0007190);adenylate cyclase-activating G-protein coupled receptor signaling pathway(GO:0007189);inflammatory response(GO:0006954);positive regulation of cytosolic calcium ion concentration(GO:0007204);regulation of adenylate cyclase activity(GO:0045761);regulation of cAMP-mediated signaling(GO:0043949)	G-protein coupled receptor(PC00021)
CACTIN	2,584,873-2,596,654	-	-	-
PIP5K1C	2,598,300-2,646,780	kinase activity(GO:0016301)	G-protein coupled receptor signaling pathway(GO:0007186);cell surface receptor signaling pathway(GO:0007166);phospholipid metabolic process(GO:0006644)	kinase(PC00137)
TJP3	2,650,749-2,674,599	-	anatomical structure morphogenesis(GO:0009653);cellular process(GO:0009987)	tight junction(PC00214)
APBA3	2,674,686-2,680,655	amyloid-beta binding(GO:0001540)	chemical synaptic transmission(GO:0007268)	membrane trafficking regulatory protein(PC00151)
MRPL54	2,680,956-2,684,815	structural constituent of ribosome(GO:0003735)	-	-
RAX2	2,684,672-2,690,012	-	-	-
MATK	2,690,173-2,696,649	-	-	-

ZFR2	2,707,832-2,745,996	double-stranded RNA binding(GO:0003725);single-stranded RNA binding(GO:0003727)	-	mRNA processing factor(PC00147)
ATCAY	2,752,177-2,779,255	pyrophosphatase activity(GO:0016462)	apoptotic signaling pathway(GO:0097190);bleb assembly(GO:0032060);cellular catabolic process(GO:0044248);execution phase of apoptosis(GO:0097194);organic substance catabolic process(GO:1901575);oxoacid metabolic process(GO:0043436);phosphorus metabolic process(GO:0006793)	-
NMRK2	2,784,101-2,788,012	kinase activity(GO:0016301)	-	glycosyltransferase(PC00111);nucleotide kinase(PC00172)
DAPK3	2,799,980-2,812,613	ATP binding(GO:0005524);protein serine/threonine kinase activity(GO:0004674)	-	non-receptor serine/threonine protein kinase(PC00167)
EEF2	2,817,111-2,825,818	GTPase activity(GO:0003924);RNA binding(GO:0003723);ribosome binding(GO:0043022)	formation of translation initiation ternary complex(GO:0001677);translational elongation(GO:0006414);translational termination(GO:0006415)	G-protein(PC00020); hydrolase(PC00121);translation elongation factor(PC00222);translation initiation factor(PC00224)
SNORD37	2,822,701-2,822,765	-	-	-

PIAS4	2,837,427-2,860,130	-	-	-
ZBTB7A	2,861,324-2,881,348	-	cellular response to DNA damage stimulus(GO:0006974)	KRAB box transcription factor(PC00029)
LOC111774179	2,895,699-2,898,590	-	-	-
MAP2K2	2,899,635-2,921,814	MAP kinase activity(GO:0004707);MAP kinase kinase activity(GO:0004708)	stress-activated protein kinase signaling cascade(GO:0031098)	-
CREB3L3	2,922,141-2,952,809	-	nervous system process(GO:0050877)	-
SIRT6	2,953,476-2,961,742	-	chromatin organization(GO:0006325)	-
ANKRD24	2,975,294-2,990,922	-	-	-
EBI3	2,991,721-2,997,888	cytokine binding(GO:0019955);cytokine receptor activity(GO:0004896)	-	cytokine(PC00083);defense/immunity protein(PC00090)
YJU2/CCDC94	2,999,351-3,014,881	catalytic activity(GO:0003824);mRNA binding(GO:0003729)	mRNA splicing, via spliceosome(GO:0000398)	mRNA splicing factor(PC00148)
LOC111774180	3,009,563-3,011,847	-	-	-
SHD	3,020,287-3,026,032	protein binding(GO:0005515)	-	-
TMIGD2	3,026,101-3,033,214	-	immune response(GO:0006955)	immunoglobulin(PC00123)
FSD1	3,033,614-3,045,559	-	-	-

STAP2	3,045,763-3,052,986	transmembrane receptor protein kinase activity(GO:0019199)	-	protein kinase receptor(PC00194)
MPND	3,060,738-3,069,336	DNA binding(GO:0003677);DNA-binding transcription factor activity(GO:0003700);chromatin binding(GO:0003682);transcription coregulator activity(GO:0003712)	regulation of transcription by RNA polymerase II(GO:0006357);transcription by RNA polymerase II(GO:0006366)	chromatin/chromatin-binding protein(PC00077);metalloprotease(PC00153);transcription cofactor(PC00217)
SH3GL1	3,069,607-3,099,353	-	-	-
MIR8996	3,127,873-3,128,007	-	-	-
UBXN6	3,129,697-3,140,713	-	-	-
HDGFL2	3,151,016-3,171,126	DNA binding(GO:0003677);DNA-binding transcription factor activity(GO:0003700);growth factor activity(GO:0008083);transcription coregulator activity(GO:0003712)	cell-cell signaling(GO:0007267);regulation of transcription by RNA polymerase II(GO:0006357);signal transduction(GO:0007165);transcription by RNA polymerase II(GO:0006366)	growth factor(PC00112);transcription cofactor(PC00217)
PLIN4	3,171,051-3,182,604	-	-	-
PLIN5	3,184,823-3,193,889	-	lipid metabolic process(GO:0006629)	transfer/carrier protein(PC00219)

LRG1	3,194,022-3,196,494	-	-	-
SEMA6B	3,199,669-3,223,797	receptor ligand activity(GO:0048018);signaling receptor activity(GO:0038023)	ameboidal-type cell migration(GO:0001667);animal organ development(GO:0048513);axon extension(GO:0048675);axon guidance(GO:0007411);cell surface receptor signaling pathway(GO:0007166);negative regulation of cell growth(GO:0030308);negative regulation of neuron projection development(GO:0010977);negative regulation of response to external stimulus(GO:0032102);positive regulation of cell migration(GO:0030335);regulation of axonogenesis(GO:0050770);regulation of cell size(GO:0008361);regulation of chemotaxis(GO:0050920);tissue development(GO:0009888)	membrane-bound signaling molecule(PC00152)
TNFAIP8L1	3,250,870-3,261,857	-	-	-
MIR8987	3,259,609-3,259,711	-	-	-
MYDGF	(3,263,269-3,274,260	-	-	-
DPP9	3,279,638-3,317,330	peptidase activity(GO:0008233)	protein acetylation(GO:0006473)	serine protease(PC00203)

TRNAG-UCC	3,317,565-3,317,636	transfer RNA glycine (anticodon UCC)	-	-
LOC106783343	3,375,904-3,378,211	-	-	-
MIR7-2	3,377,222-3,377,305	-	-	-
FEM1A	3,389,992-3,394,025	-	-	-
TICAM1	3,401,162-3,411,521	-	-	-
LOC111774185	3,403,093-3,407,098	-	-	-
PLIN3	3,416,527-3,432,418	-	lipid metabolic process(GO:0006629)	transfer/carrier protein(PC00219)
ARRDC5	3,444,760-3,454,186	-	-	-
LOC106783359	3,454,275-3,454,811	-	-	-
UHRF1	3,456,772-3,487,264	ubiquitin protein ligase activity(GO:0061630)	DNA metabolic process(GO:0006259);macromolecule methylation(GO:0043414)	ubiquitin-protein ligase(PC00234)
TIF-2-like	3,491,811-3,496,261	-	-	-

KDM4B	3,492,192-3,611,468	histone demethylase activity(GO:0032452);oxidoreductase activity, acting on paired donors, with incorporation or reduction of molecular oxygen, 2-oxoglutarate as one donor, and incorporation of one atom each of oxygen into both donors(GO:0016706)	chromatin remodeling(GO:0006338)	zinc finger transcription factor(PC00244)
MIR8982	3,592,293-3,592,431	-	-	-
PTPRS	3,647,749-3,742,796	phosphoprotein phosphatase activity(GO:0004721)	-	protein phosphatase(PC00195)
ZNRF4	3,805,230-3,806,826	ubiquitin protein ligase activity(GO:0061630)	ubiquitin-dependent protein catabolic process(GO:0006511)	-
LOC102149040	3,807,004-3,808,483	-	-	-
SAFB2	3,866,633-3,896,833	sequence-specific DNA binding(GO:0043565)	mRNA processing(GO:0006397);regulation of mRNA processing(GO:0050684);regulation of transcription by RNA polymerase II(GO:0006357);transcription by RNA polymerase II(GO:0006366)	-

SAFB	3,896,769-3,927,857	sequence-specific DNA binding(GO:0043565)	intracellular steroid hormone receptor signaling pathway(GO:0030518);mRNA processing(GO:0006397);regulation of mRNA processing(GO:0050684);regulation of transcription by RNA polymerase II(GO:0006357);transcription by RNA polymerase II(GO:0006366)	-
MICOS13	3,931,486-3,933,610	-	-	-
HSD11B1L	3,933,762-3,939,029	-	-	-
RPL36	3,940,102-3,941,132	structural constituent of ribosome(GO:0003735)	cytoplasmic translation(GO:0002181);formation of translation initiation ternary complex(GO:0001677);translational elongation(GO:0006414);translational termination(GO:0006415)	ribosomal protein(PC00202)
LONP1	3,941,182-3,959,157	ATPase activity, coupled(GO:0042623);peptidase activity, acting on L-amino acid peptides(GO:0070011);single-stranded DNA binding(GO:0003697)	cellular protein-containing complex assembly(GO:0034622);mitochondrion organization(GO:0007005);protein quality control for misfolded or incompletely synthesized proteins(GO:0006515)	serine protease(PC00203)
LOC111774190	3,942,342-3,943,167	-	-	-
CATSPERD	3,956,874-3,998,757	-	-	-

PRR22	4,001,165-4,003,176	-	-	-
MIR8991	4,005,902-4,006,046	-	-	-
NRTN	4,015,883-4,027,325	cytokine activity(GO:0005125);growth factor activity(GO:0008083)	cellular process(GO:0009987)	neurotrophic factor(PC00163)
FUT3-like	4,029,829-4,044,992	-	-	-
NDUFA11	4,049,001-4,056,121	-	-	-
VMAC	4,056,380-4,061,225	-	-	-
CAPS	4,061,243-4,063,479	-	-	-
LOC111774193	4,067,497-4,068,463	-	-	-
RANBP3	4,064,447-4,115,864	GTPase activator activity(GO:0005096);GTPase activity(GO:0003924);Ras GTPase binding(GO:0017016)	protein export from nucleus(GO:0006611)	G-protein modulator(PC00022)
LOC111774194	4,115,915-4,117,228	-	-	-

RFX2	4,125,487-4,217,688	DNA-binding transcription factor activity(GO:0003700);RNA polymerase II proximal promoter sequence-specific DNA binding(GO:0000978)	regulation of transcription by RNA polymerase II(GO:0006357);transcription by RNA polymerase II(GO:0006366)	winged helix/forkhead transcription factor(PC00246)
LOC111774195	4,192,165-4,196,939	-	-	-
ACSBG2	4,238,170-4,269,643	ligase activity(GO:0016874);transporter activity(GO:0005215)	fatty acid metabolic process(GO:0006631);immune system process(GO:0002376);lipid transport(GO:0006869)	ligase(PC00142)
LOC111774196	4,268,739-4,279,591	-	-	-
MLLT1	4,296,102-4,354,138	DNA binding(GO:0003677);DNA-binding transcription factor activity(GO:0003700);chromatin binding(GO:0003682)	transcription by RNA polymerase II(GO:0006366)	chromatin/chromatin-binding protein(PC00077);transcription factor(PC00218)
ACER1	4,362,036-4,379,243	-	immune system process(GO:0002376)	-
CLPP	4,401,105-4,405,412	ATPase activity, coupled(GO:0042623);enzyme binding(GO:0019899);serine-type endopeptidase activity(GO:0004252)	protein quality control for misfolded or incompletely synthesized proteins(GO:0006515)	-

ALKBH7	4,407,210-4,408,946	-	-	-
PSPN	4,409,059-4,412,334	cytokine activity(GO:0005125);growth factor activity(GO:0008083)	cellular process(GO:0009987)	neurotrophic factor(PC00163)
GTF2F1	4,412,979-4,423,086	DNA binding(GO:0003677);DNA-binding transcription factor activity(GO:0003700)	transcription elongation from RNA polymerase II promoter(GO:0006368);transcription initiation from RNA polymerase II promoter(GO:0006367)	nucleic acid binding(PC00171);transcription factor(PC00218)
AK1	4,422,797-4,438,872	kinase activity(GO:0016301)	pyrimidine nucleobase metabolic process(GO:0006206)	nucleotide kinase(PC00172)
KHSRP	4,438,465-4,449,545	catalytic activity(GO:0003824);mRNA binding(GO:0003729);protein binding(GO:0005515)	apoptotic process(GO:0006915);intracellular protein transport(GO:0006886);mRNA splicing, via spliceosome(GO:0000398);nervous system process(GO:0050877);nuclear transport(GO:0051169);protein metabolic process(GO:0019538);signal transduction(GO:0007165);transcription by RNA polymerase II(GO:0006366)	enzyme modulator(PC00095);mRNA splicing factor(PC00148);ribonucleoprotein(PC00201);serine protease(PC00203)

SLC25A41	4,450,003-4,457,935	ATP transmembrane transporter activity(GO:0005347)	-	amino acid transporter(PC00046); calmodulin(PC00061); mitochondrial carrier protein(PC00158); transfer/carrier protein(PC00219)
SLC25A23	4,458,510-4,472,785	ATP transmembrane transporter activity(GO:0005347)	-	amino acid transporter(PC00046); calmodulin(PC00061); mitochondrial carrier protein(PC00158); transfer/carrier protein(PC00219)
CRB3	4,473,525-4,477,765	-	-	-
DENND1C	4,477,763-4,486,986	GDP binding(GO:0019003);GTP binding(GO:0005525);Rab guanyl-nucleotide exchange factor activity(GO:0017112);phosphatidylinositol phosphate binding(GO:1901981)	endocytic recycling(GO:0032456);endocytosis(GO:0006897);membrane invagination(GO:0010324);vesicle budding from membrane(GO:0006900)	-
TUBB4A	4,492,410-4,497,646	GTP binding(GO:0005525);str	microtubule cytoskeleton organization(GO:0000226);mitotic nuclear division(GO:0140014)	tubulin(PC00228)

		uctural molecule activity(GO:0005198)		
TNFSF9	4,512,344-4,516,100	cytokine receptor binding(GO:0005126)	T cell differentiation(GO:0030217);T cell proliferation(GO:0042098);leukocyte cell-cell adhesion(GO:0007159);positive regulation of T cell proliferation(GO:0042102);positive regulation of cell differentiation(GO:0045597);positive regulation of multicellular organismal process(GO:0051240);regulation of lymphocyte differentiation(GO:0045619)	-
CD70	4,579,851-4,583,253	-	-	-
TNFSF14/TNLG1D	4,654,506-4,658,932	cytokine activity(GO:0005125)	apoptotic process(GO:0006915);cell-cell signaling(GO:0007267);cytokine-mediated signaling pathway(GO:0019221);immune system process(GO:0002376)	tumor necrosis factor family member(PC00229)
C3-like	4,664,938-4,705,436	-	-	-
C3	4,732,581-4,764,235	cytokine activity(GO:0005125);peptidase inhibitor activity(GO:0030414)	cellular process(GO:0009987);proteolysis(GO:0006508);response to stimulus(GO:0050896)	complement component(PC00078);cytokine(PC00083);serine protease inhibitor(PC00204)

GPR108	4,771,857-4,779,490	G-protein coupled receptor activity(GO:0004930)	-	G-protein coupled receptor(PC00021)
TRIP10	4,781,537-4,790,073	-	-	-
VAV1	4,803,149-4,859,335	-	G-protein coupled receptor signaling pathway(GO:0007186);JNK cascade(GO:0007254);calcium-mediated signaling(GO:0019722);cell surface receptor signaling pathway(GO:0007166);immune system process(GO:0002376);nervous system process(GO:0050877)	-
ADGRE1	4,879,609-4,948,789	G-protein coupled receptor activity(GO:0004930);adenylate cyclase activity(GO:0004016)	activation of adenylate cyclase activity(GO:0007190);adenylate cyclase-activating G-protein coupled receptor signaling pathway(GO:0007189);regulation of adenylate cyclase activity(GO:0045761);regulation of cAMP-mediated signaling(GO:0043949)	G-protein coupled receptor(PC00021);antibacterial response protein(PC00051);protease(PC00190)
ADGRE4P-putatitve	4,973,516-5,013,512	-	-	-

Table S2: Horses used in qPCR analysis. Age was not significantly different between disease statuses based on a Student's t-test. Abbreviations: Breeds - QH = Quarter Horse, POA = Pony of the Americas, TB = Thoroughbred, Status - U = unaffected control, eNAD/EDM= equine neuroaxonal dystrophy/degenerative myeloencephalopathy and Tissue - C1 = Cervical spinal cord at level of C1 vertebrae

Horse #	Age (y)	Sex	Breed	Status	Tissue
1	1	Mare	QH	U	C1
2	3	Mare	QH	eNAD/EDM	Brainstem
3	1	Stallion	QH	eNAD/EDM	Brainstem
4	2	Gelding	QH	U	Brainstem
5	1.5	Stallion	QH	U	Brainstem
6	1.5	Mare	QH	eNAD	Brainstem
7	4	Gelding	QH	eNAD/EDM	Brainstem
8	1.5	Mare	QH	eNAD/EDM	Brainstem
9	4	Mare	QH	eNAD/EDM	Brainstem
10	1	Mare	POA	U	Brainstem
11	1	Stallion	QH	eNAD/EDM	C1
12	1	Gelding	Shire	eNAD/EDM	Brainstem
13	5	Gelding	TB	U	Brainstem
14	2	Mare	QH	U	Brainstem
15	6	Mare	QH	U	Brainstem

Table S3: Mice used in the histological study. Individuals highlighted by red text were used for Iba1 staining.

	Genotype	Diet	Sex	
1 month	*Atcay*$^{+/+}$	Basal	♂	n=2
			♀	n=2
	Atcay$^{hes/hes}$	Basal	♂	n=2
			♀	n=2
2 month	*Atcay*$^{+/+}$	Basal	♂	n=2
			♀	n=2
	Atcay$^{hes/hes}$	Basal	♂	n=2
			♀	n=2
1 month	*Atcay*$^{+/+}$	Supp.	♂	n=2
			♀	n=2
	Atcay$^{hes/hes}$	Supp.	♂	n=2
			♀	n=1
2 month	*Atcay*$^{+/+}$	Supp.	♂	n=2
			♀	n=2
	Atcay$^{hes/hes}$	Supp.	♂	n=2
			♀	n=2

Table S4: Conversion between genome builds. The position of the SNPs that passed FDR correction, as well as three candidate genes in the EquCab2.0 and EquCab3.0 genome build. Lift over created using NCBI remap tool (https://www.ncbi.nlm.nih.gov/genome/tools/remap).

Location Identifier	EquCab2.0 Position	EquCab3.0 Position	*P*-value
ZNF77	1,555,110-1,572,205	1,942,392-1,959,392	N/A
AX-102952090	1,712,608	2,092,035	4.720856×10^{-6}
AX-103851457	1,788,729	2,168,012	2.050603×10^{-7}
ATCAY	2,334,345-2,353,861	2,752,177-2,779,255	N/A
AX-104814008	3,499,949	3,977,658	1.11537×10^{-6}
AX-103878828	3,717,996	4,194,129	1.127115×10^{-6}
ACSBG2	3,762,099-3,793,519	4,238,170-4,269,643	N/A
AX-104272534	4,048,090	4,522,477	9.487773×10^{-7}
AX-104841663	4,051,156	4,525,543	9.487773×10^{-7}
AX-104596120	4,053,961	4,528,348	7.695151×10^{-8}

Table S5: Variants within the 2.5 Mb region on ECA7 identified using WGS data and genotyped using MassArray® platform. Predicted effect on protein was evaluated using SNP_EFF. *P* value based on Wald test from GEMMA. Variants listed as "Not Tested" failed QC and were filtered out.

Position	SNP_EFF Annotation	Reference	Alternate	Selection Reason	*P* Wald
1,689,643	Intronic GNG7	C	T	Intragenic	0.1653356
1,820,066	NA	G	A	Representative of a haplotype	0.000801401
1,857,584	Intronic ZNF544	C	T	Intragenic	9.68412E-05
1,861,908	3' ZNF544	G	T	Representative of a haplotype	0.01609736
1,869,777	5' ZNF555	G	A	Close to coding region	0.001434306
1,870,811	5' ZNF555	C	G	Close to coding region	0.000801401
1,871,637	5' ZNF555	C	T	Close to coding region	0.000801401
1,873,376	5' ZNF555	G	A	Close to coding region	0.01609736
1,873,435	5' ZNF555	A	G	Close to coding region	0.09303797
1,880,061	NA	C	G	Representative of a haplotype	0.002879717
1,908,847	Missense ZNF556	C	T	Missense	0.000673946
1,909,412	3' ZNF556	C	T	Close to coding region	0.01118315
1,927,530	NA	A	G	Representative of a haplotype	0.01609736
1,954,155	5' LOC100060110	G	T	Close to coding region	0.01609736
1,967,686	NA	C	A	Representative of a haplotype	0.01609736
1,994,821	Intronic LOC100629730	T	C	Close to coding region	0.01609736
2,019,560	NA	C	T	Representative of a haplotype	0.01609736
2,036,473	Intronic LOC	A	G	Close to coding region	Failed QC
2,055,095	Downstream LOC	C	A	Representative of a haplotype	Failed QC
2,055,848	NA	A	G	Representative of a haplotype	0.01609736
2,056,240	NA	C	T	Representative of a haplotype	0.01984063
2,056,657	NA	G	A	Representative of a haplotype	Failed QC
2,062,332	Intronic LOC100060011	C	T	Representative of a haplotype	0.01609736
2,062,776	Intronic LOC111774434	GTGAA	CTGAG	Representative of a haplotype	0.3894071

2,062,988	Intronic LOC111774434	G	A	Representative of a haplotype	0.2732147
2,063,268	Intronic LOC111774434	T	C	Representative of a haplotype	0.01896806
2,064,768	Intronic LOC111774434	C	T	Representative of a haplotype	0.01609736
2,065,221	Intronic LOC111774434	A	G	Representative of a haplotype	0.01609736
2,065,762	NA	A	G	Representative of a haplotype	0.01609736
2,066,097	NA	A	G	Representative of a haplotype	0.01609736
2,066,242	NA	C	A	Representative of a haplotype	0.01609736
2,070,967	Missense LOC111774137	G	C	Missense	Failed QC
2,073,010	NA	G	A	Representative of a haplotype	Failed QC
2,077,256	NA	C	T	Representative of a haplotype	0.01609736
2,090,771	Intronic LOC102148548	C	T	Close to coding region	2.20539E-05
2,092,257	Intronic LOC102148548	G	A	Close to coding region	4.72086E-06
2,092,896	NA	G	C	Representative of a haplotype	Failed QC
2,093,995	Coding LOC106783362	G	A	Coding	4.72086E-06
2,094,447	Coding LOC106783362	G	T	Coding	Failed QC
2,095,100	Coding LOC106783362	A	G	Coding	0.01038143
2,095,144	Coding LOC106783362	CAGA	CA	Coding	Failed QC
2,095,151	Coding LOC106783362	C	T	Coding	0.1834841
2,095,674	Coding LOC100059925 and coding LOC106783362	T	C	Coding	0.01609736
2,098,665	Coding LOC100059925 and Intronic LOC106783362	TACAACAA	TACAA	Coding	Failed QC
2,100,530	Coding LOC100059925 and Intronic LOC106783362	T	C	Coding	1.5661E-07
2,100,683	Coding LOC100059925 and Intronic LOC106783362	A	G	Coding	1.63882E-05
2,100,814	Coding LOC100059925 and Intronic LOC106783362	T	G	Coding	1.5661E-07
2,101,231	Coding LOC100059925 and Intronic LOC106783362	A	G	Coding	1.5661E-07

2,101,286	Coding LOC100059925 and Intronic LOC106783362	C	T	Coding	1.5661E-07
2,101,711	Missense LOC	T	C	Missense	1.5661E-07
2,102,193	Intronic LOC100059925 and Intronic LOC106783362	G	A	Close to coding region	1.5661E-07
2,104,377	Intronic LOC100059925	A	G	Close to coding region	1.5661E-07
2,107,635	Intronic LOC100059925	G	T	Close to coding region	1.5661E-07
2,112,501	NA	A	G	Representative of a haplotype	0.01609736
2,124,326	NA	G	A	Representative of a haplotype	0.01609736
2,128,750	NA	G	A	Representative of a haplotype	0.01609736
2,132,141	NA	G	T	Representative of a haplotype	0.01609736
2,136,019	NA	G	C	Representative of a haplotype	0.01609736
2,136,523	NA	G	C	Representative of a haplotype	0.01609736
2,154,133	Intergenic	G	A	Representative of a haplotype	Failed QC
2,154,470	Intergenic	C	T	Representative of a haplotype	0.00012909
2,157,908	Intergenic	C	T	Representative of a haplotype	Failed QC
2,164,319	Intergenic	G	A	Representative of a haplotype	0.1653356
2,227,569	Intronic AES	G	A	Representative of a haplotype	0.000623936
2,236,972	Intergenic	A	G	Representative of a haplotype	0.1474469
2,290,405	Intronic GNA15	G	T	Representative of a haplotype	0.01622388
2,290,900	Intronic GNA15	A	G	Representative of a haplotype	0.01622388
2,299,240	Intronic GNA15	C	T	Representative of a haplotype	0.007541493
2,320,091	Intronic NCLN- middle	C	T	Representative of a haplotype	0.005323477
2,329,926	NCLN 3'UTR	C	T	Representative of a haplotype	Failed QC
2,332,203	NCLN 3'UTR	C	T	Representative of a haplotype	0.01049127
2,381,821	Downstream CELF5	C	T	Close to coding region	0.01456
2,384,479	Downstream CELF5	C	G	Close to coding region	0.01456
2,384,527	Downstream CELF5	T	C	Close to coding region	Failed QC
2,384,574	Downstream CELF5	G	T	Close to coding region	Failed QC
2,404,995	Downstream CELF5	G	T	Close to coding region	Failed QC

2,591,165	Intronic CACTIN	C	T	Representative of a haplotype	Failed QC
2,596,696	Upstream CACTIN	A	G	Representative of a haplotype	0.1918071
2,599,169	downstream PIP5K1C	C	T	Representative of a haplotype	0.01175034
2,655,987	Upstream TJP3	C	T	Representative of a haplotype	0.05125067
2,709,102	Downstream ZFR2	G	A	Representative of a haplotype	Failed QC
2,720,898	Synonymous ZFR2	C	T	Coding	0.7570154
2,720,943	Synonymous ZFR2	C	G	Coding	Failed QC
2,720,944	Missense ZFR2	C	T	Missense	Failed QC
2,726,752	Intronic ZFR2	G	T	Close to coding region	Failed Design
2,755,141	Synonymous ATCAY	C	T	Coding	0.1708744
2,769,147	Intronic ATCAY	C	T	Gene of interest	0.03504549
2,770,991	Intronic ATCAY	A	T	Gene of interest	0.000623936
2,807,687	Intronic DAPK3	C	T	Close to coding region	0.000623936
2,835,772	Upstream PIAS4	G	A	Close to coding region	Failed QC
2,922,148	Missense CREB3L3	C	T	Missense	Failed QC
2,938,439	Intronic CREB3L3	A	G	Representative of a haplotype	0.01726911
3,013,620	NA	G	T	Representative of a haplotype	Failed QC
3,334,329	NA	C	A	Representative of a haplotype	0.1322512
3,480,812	Intronic UHRF1	G	C	Representative of a haplotype	0.4039229
3,486,593	3' UHRF1	C	T	Representative of a haplotype	0.09297081
3,511,215	Intronic KDMB4	A	G	Representative of a haplotype	Failed QC
3,545,781	Intronic KDMB4	C	T	Representative of a haplotype	0.3189479
3,587,618	NA	T	G	Representative of a haplotype	0.6699477
3,595,408	Intronic KDMB4	C	T	Representative of a haplotype	0.2185643
3,596,189	Intronic KDMB4	G	A	Representative of a haplotype	0.1711823
3,611,974	Downstream KDMB4	C	T	Representative of a haplotype	0.1601115
3,730,789	Intronic PTPRS	A	G	Representative of a haplotype	0.003045052
3,778,956	NA	G	A	Representative of a haplotype	0.5365844
3,779,978	NA	A	G	Representative of a haplotype	0.4615653
3,791,505	NA	A	G	Representative of a haplotype	0.7394344

3,807,756	NA	G	A	Representative of a haplotype	0.003967438
3,808,672	NA	C	T	Representative of a haplotype	0.003967438
3,817,029	NA	C	T	Representative of a haplotype	0.2401019
3,817,547	NA	A	G	Representative of a haplotype	0.8265041
3,831,537	NA	C	A	Representative of a haplotype	0.006661491
3,833,608	NA	C	T	Representative of a haplotype	0.3863172
3,835,064	NA	C	T	Representative of a haplotype	0.00807628
3,842,109	NA	G	C	Representative of a haplotype	Failed QC
3,871,275	Downstream SAFB2	G	A	Close to coding region	5.05232E-05
3,890,969	Missense SAFB2	G	A	Missense	5.05232E-05
3,894,116	Downstream SAFB2	G	A	Close to coding region	5.05232E-05
3,900,566	Downstream SAFB2	G	A	Close to coding region	5.05232E-05
3,940,002	NA	C	A	Representative of a haplotype	0.01320134
3,951,726	Intronic LONP1	C	T	Representative of a haplotype	0.00070136
3,957,141	5' CATSPERD	C	T	Close to coding region	0.04594766
3,957,204	5' CATSPERD	C	T	Close to coding region	0.2830304
3,957,237	5' CATSPERD	A	G	Close to coding region	0.007031986
3,957,254	5' CATSPERD	T	C	Close to coding region	0.008838997
3,964,121	Intronic CATSPERD	C	A	Representative of a haplotype	0.000262669
3,974,742	Intronic CATSPERD	C	T	Representative of a haplotype	5.05232E-05
3,983,765	Intronic CATSPERD	T	C	Representative of a haplotype	0.1653356
4,006,799	NA	G	C	Representative of a haplotype	Failed QC
4,023,439	Intronic NRTN	C	T	Representative of a haplotype	2.95825E-07
4,027,229	3' NRTN	C	T	Close to coding region	Failed QC
4,029,537	3' NRTN	C	T	Close to coding region	1.54783E-07
4,031,004	Missense LOC	C	T	Missense	1.54783E-07
4,110,180	Intronic RANBP3	C	T	Representative of a haplotype	0.001520387
4,118,545	Upstream RANBP3	C	T	Close to coding region	0.001520387
4,149,335	Intronic RFX2	A	C	Representative of a haplotype	1.12712E-06
4,154,300	Intronic RFX2	T	C	Representative of a haplotype	1.12712E-06

4,174,303	Intronic RFX2	A	C	Representative of a haplotype	1.12712E-06
4,194,440	Intronic RFX2	G	A	Representative of a haplotype	0.04639769
4,199,703	Intronic RFX2	G	A	Representative of a haplotype	0.2260572
4,203,198	Intronic RFX2	T	C	Representative of a haplotype	1.12712E-06
4,239,946	Upstream ACSBG2	G	A	Close to coding region	1.05148E-05
4,254,275	Intronic ACSBG2	C	T	Representative of a haplotype	8.98578E-06
4,257,492	Intronic ACSBG2	C	T	Representative of a haplotype	1.91366E-05
4,266,121	3' ACSBG2	T	A	Close to coding region	8.98578E-06
4,268,909	3' ACSBG2	C	T	Close to coding region	0.01280451
4,269,320	3' ACSBG2	T	C	Close to coding region	0.01280451
4,269,726	3' ACSBG2	G	A	Close to coding region	8.98578E-06
4,270,115	3' ACSBG2	A	C	Close to coding region	8.98578E-06
4,270,352	3' ACSBG2	G	A	Close to coding region	Failed QC
4,272,041	3' ACSBG2	G	A	Close to coding region	8.98578E-06
4,272,345	3' ACSBG2	G	A	Close to coding region	0.000165106
4,272,470	3' ACSBG2	G	A	Close to coding region	8.98578E-06
4,273,102	3' ACSBG2	G	A	Close to coding region	5.21141E-05
4,274,052	3' ACSBG2	T	C	Close to coding region	8.98578E-06
4,275,994	NA	A	C	Representative of a haplotype	0.1653356
4,286,887	NA	G	A	Representative of a haplotype	0.01280451
4,294,044	Downstream MLLT1	G	A	Close to coding region	0.04886884
4,328,825	Intronic MLLT1	C	T	Representative of a haplotype	8.98578E-06
4,347,579	Intronic MLLT1	C	T	Representative of a haplotype	8.98578E-06
4,361,496	Downstream MLLT1	T	G	Close to coding region	4.91453E-05
4,362,074	3' ACER1	G	A	Close to coding region	0.000134275
4,362,214	3' ACER1	C	T	Close to coding region	0.003220997
4,364,960	Intronic ACER1	C	A	Representative of a haplotype	0.000134275
4,413,860	NA	C	A	Representative of a haplotype	0.002658613
4,416,327	NA	C	T	Representative of a haplotype	Failed QC
4,422,787	5' GTF2F1	C	T	Close to coding region	0.7181657

4,422,817	5' GTF2F1	T	G	Close to coding region	0.7524691
4,423,173	5' GTF2F1	C	G	Close to coding region	0.3864939
4,456,871	5' SLC25A41	G	A	Close to coding region	0.002658613
4,470,527	Intronic SLC25A41	G	A	Representative of a haplotype	2.15146E-05
4,496,353	Intronic TUBB4A	G	T	Representative of a haplotype	5.63382E-05
4,518,740	Downstream TNFSF9	G	A	Close to coding region	0.000387576
4,519,638	Downstream TNFSF9	T	G	Close to coding region	0.01017786
4,519,750	Downstream TNFSF9	A	G	Close to coding region	0.01017786
4,520,124	Downstream TNFSF9	C	T	Close to coding region	0.01017786
4,520,290	Downstream TNFSF9	G	A	Close to coding region	0.718416
4,520,979	Downstream TNFSF9	T	A	Close to coding region	0.01017786
4,521,325	NA	C	A	Representative of a haplotype	0.01503226
4,522,905	NA	A	T	Representative of a haplotype	0.01017786
4,523,670	NA	G	C	Representative of a haplotype	Failed QC
4,524,206	NA	C	A	Representative of a haplotype	0.01017786
4,526,401	NA	A	G	Representative of a haplotype	Failed QC
4,528,348	NA	A	G	Representative of a haplotype	9.48777E-07
4,534,990	NA	G	A	Representative of a haplotype	9.48777E-07
4,538,050	NA	T	C	Representative of a haplotype	9.48777E-07
4,576,330	Downstream CD70	A	G	Close to coding region	4.45443E-05
4,718,226	NA	T	C	Representative of a haplotype	0.006738345
4,779,405	Missense GRP108	G	C	Missense	0.02483918
4,851,041	Intronic VAV1	G	A	Representative of a haplotype	Failed QC
4,861,792	Downstream VAV1	A	T	Close to coding region	0.02829077
4,863,316	Downstream VAV1	G	T	Close to coding region	0.005464644
4,893,405	Intronic ADGRE1	A	C	Close to coding region	Failed QC
4,917,144	Intronic ADGRE1	G	T	Close to coding region	Failed QC
4,917,728	Intronic ADGRE1	C	T	Close to coding region	0.02777557
4,928,733	Missense ADGRE1	A	C	Missense	0.1379559
4,948,983	Downstream ADGRE1	G	A	Close to coding region	0.1967953

4,988,097	Intronic LOC	A	T	Representative of a haplotype	0.2445771
4,990,279	Intronic LOC	A	G	Representative of a haplotype	0.4390001
5,019,588	Intronic LOC	T	C	Representative of a haplotype	0.4390001
5,024,838	Missense LOC	T	C	Missense	0.7046878

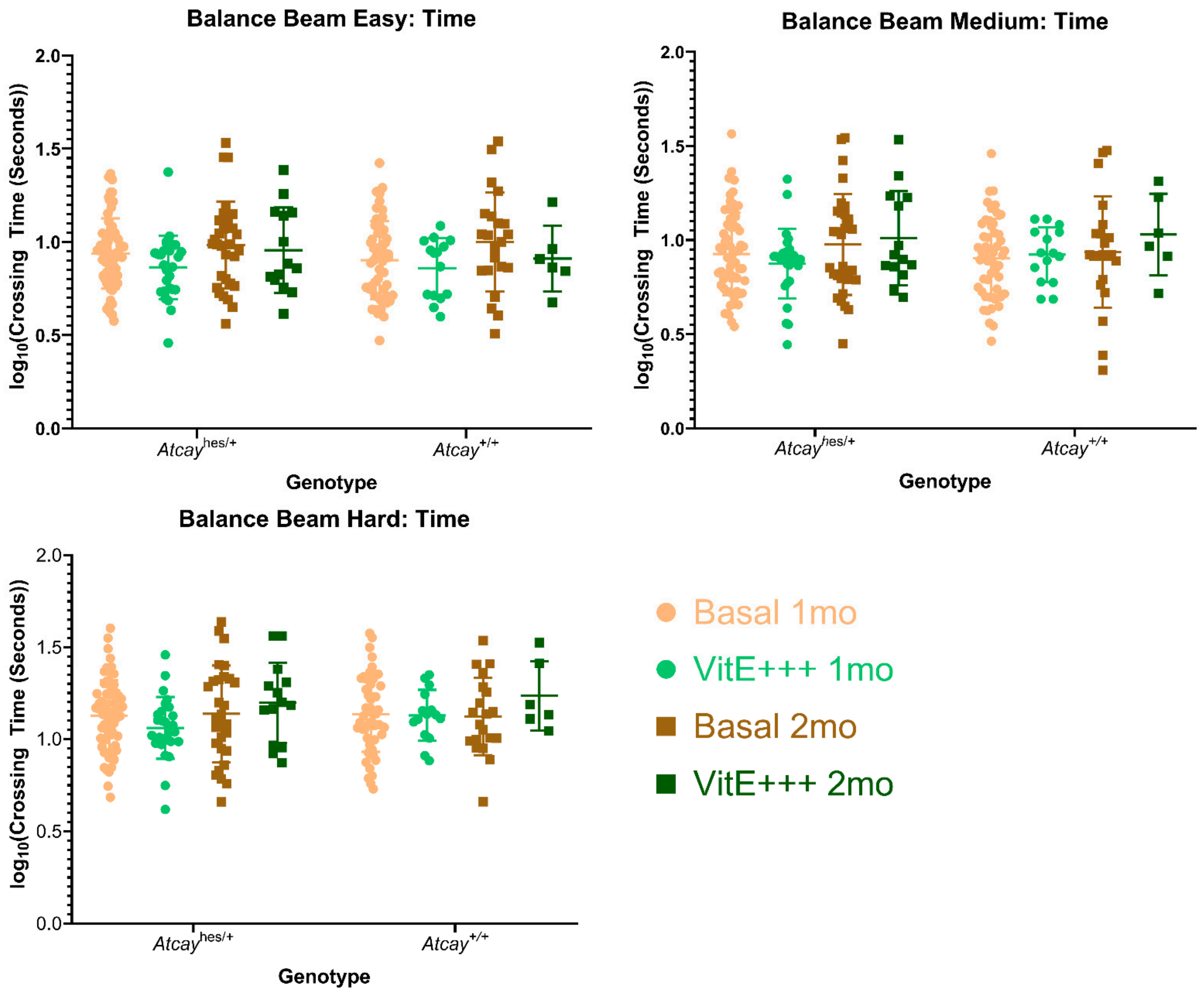

Figure S1: Balance beam data showing no changes for the *Atcay*ji-hes mouse. WGS raw sequenced data available on the SRA database under Accession number PRJNA526073.
